# Resumption of Peritoneal Dialysis Postpartum Following Pregnancy in an ESRD Patient: A Case Report and Literature Review

**DOI:** 10.1002/ccr3.71266

**Published:** 2025-10-12

**Authors:** Ahmad Matarneh, Sundus Sardar, Omar Salameh, Abdelrauof Akkari, Umar Farooq, Navin Verma, Nasrollah Ghahramani, Ronald Miller

**Affiliations:** ^1^ Division of Nephrology, Department of Medicine Penn State Health, Milton S. Hershey Medical Center Hershey Pennsylvania USA; ^2^ Department of nephrology university health truman medical center Kansas City Missouri USA

**Keywords:** intermittent hemodialysis, maternal‐fetal, nephrology, peritoneal dialysis, pregnancy

## Abstract

Temporary transition from peritoneal to hemodialysis during pregnancy does not need to be permanent. When catheter patency and membrane function are preserved, peritoneal dialysis can be safely resumed postpartum. This case reinforces the importance of individualized, multidisciplinary dialysis management to optimize maternal and fetal outcomes in pregnant ESRD patients.

## 1 Introduction

Pregnancy in dialysis‐dependent women is both rare and high risk, with incidence estimates between 1 and 7 per 1000 patients annually [1]. Historically, such pregnancies were discouraged due to poor fetal outcomes, but modern dialysis strategies and multidisciplinary care have significantly improved survival and birth outcomes [2].

Hemodialysis is typically favored during pregnancy for its enhanced ability to manage volume, small solute clearance, and acid–base status. However, PD remains a viable and sometimes preferred option for selected patients due to its continuous clearance, cardiovascular stability, and preservation of residual renal function [3]. As more women of childbearing age are maintained on PD, questions about modality transitions during pregnancy and postpartum reinitiation have grown increasingly relevant.

While previous reports have described peritoneal dialysis during or after pregnancy, our case uniquely contributes by demonstrating a seamless, complication‐free reinitiation of CAPD following intensive 6‐day‐per‐week HD during pregnancy, guided by objective PET assessment. Additionally, the preservation of the PD catheter and use of functional testing before reinitiation provide a practical template for clinical decision‐making. This case adds to the limited but growing body of literature supporting individualized, modality‐flexible dialysis management in reproductive‐aged women with ESRD.

This case report outlines the successful reinitiation of PD postpartum in a woman who had temporarily transitioned to HD during pregnancy. In parallel, we review the current literature on pregnancy outcomes in patients maintained on PD, highlighting key physiological considerations, management strategies, and real‐world experiences.

## 2 Case History and Examination

A 40‐year‐old woman with end‐stage kidney disease (ESKD) secondary to biopsy‐confirmed focal segmental glomerulosclerosis (FSGS) was initiated on continuous ambulatory peritoneal dialysis (CAPD) in 2022. Her initial dialysis course was stable, with effective solute clearance (weekly Kt/V > 1.8), adequate ultrafiltration, and preserved residual urine output (> 500 mL/day). She had no history of peritonitis or catheter dysfunction.

In early 2024, the patient became pregnant. Given the increased metabolic demands of pregnancy and the improved fetal outcomes associated with intensive dialysis, she was counseled extensively by a multidisciplinary team comprising nephrology, maternal‐fetal medicine, and dialysis nursing. In the first trimester, she elected to transition to in‐center hemodialysis (HD) six times per week. The HD regimen targeted pre‐dialysis BUN < 50 mg/dL with individualized fluid removal and close monitoring of labs and fetal well‐being. Her peritoneal dialysis catheter was retained and flushed intermittently to preserve patency.

## 3 Differential Diagnosis, Investigations, and Treatment

Her pregnancy was uncomplicated, and she delivered a healthy infant at term via spontaneous vaginal delivery.

At 2 months postpartum, she expressed interest in resuming PD. A peritoneal equilibration test (PET) confirmed preserved membrane function (low‐average transporter), and assessments of peritoneal and residual renal clearance confirmed adequacy for CAPD. She was successfully restarted on her previous PD regimen: four daily exchanges using 2.5% dextrose solution.

## 4 Conclusion and Results (Outcome and Follow‐Up)

At six‐month follow‐up, the patient remained clinically stable on CAPD, with no episodes of peritonitis, catheter malfunction, or ultrafiltration failure. Her weekly Kt/V remained above 1.8, and fluid balance was well maintained with daily ultrafiltration volumes averaging 1.2–1.5 L. Residual urine output remained at approximately 400–500 mL/day. Blood pressure and volume status were stable without the need for antihypertensives or hypertonic PD solutions. The patient's preference for home‐based therapy was a central factor in the decision to resume CAPD. Her case highlights the feasibility of resuming PD postpartum following a temporary transition to HD during pregnancy.

## 5 Discussion

Pregnancy in women with ESRD undergoing dialysis poses significant physiological and clinical challenges. Expanded plasma volume, increased metabolic waste generation, and altered peritoneal membrane dynamics complicate dialysis management [4]. Hemodynamic stability, volume control, and solute clearance must be carefully balanced to optimize both maternal and fetal outcomes.

In many centers, in‐center hemodialysis (HD) is preferred during pregnancy because it allows for more aggressive and predictable solute clearance. Intensive HD, typically performed 5–6 times per week, has been associated with improved birth weights and gestational age at delivery [5]. However, frequent HD may accelerate the decline of residual renal function (RRF), contribute to vascular access complications, and impose considerable logistical burdens. In contrast, peritoneal dialysis (PD) offers continuous clearance with greater hemodynamic stability and may better preserve RRF—factors linked to improved fetal outcomes [6]. Yet, concerns about decreased clearance, mechanical complications from abdominal distention, and infection risks often prompt clinicians to transition PD patients to HD during pregnancy [7].

Our case illustrates that such transitions need are not permanent. When peritoneal membrane function is preserved, and the catheter remains in situ, postpartum resumption of PD can be safe and effective. This strategy maintains patient autonomy and avoids additional invasive procedures. In our patient, the postpartum return to PD was seamless. Several factors contributed to this success: (1) preservation of the PD catheter throughout pregnancy, (2) favorable peritoneal equilibration test (PET) results indicating low‐average transport, (3) continued urine output supporting ultrafiltration goals, and (4) strong patient motivation to resume home‐based therapy.

The most critical determinant of pregnancy outcomes in dialysis patients—regardless of modality—is achieving dialysis adequacy. A weekly Kt/V of ≥ 2.2 is recommended during pregnancy to minimize uremic toxicity and support fetal growth [8]. In PD, this often necessitates increased exchange frequency or use of automated PD. Additionally, preservation of RRF has been independently associated with higher birth weights and reduced rates of preterm delivery [9].

An increasing number of case reports and small case series now document successful pregnancies on PD, particularly when regimens are intensified and closely monitored. For example, Lim et al. [10] described a successful term pregnancy in a 42‐year‐old woman who remained on CAPD throughout gestation. Alhwiesh et al. [11] published the largest known case series of PD in pregnancy to date, involving eight women managed with tidal automated PD (APD), all of whom delivered live infants without major maternal or fetal complications.

Postpartum reinitiation of PD has also been reported. Smith et al. [12] and Nivelle et al. [13] described cases in which patients temporarily transitioned to HD during pregnancy and resumed PD afterward without complications. These findings support the notion that PD can remain a viable long‐term strategy even when interrupted for pregnancy‐related reasons.

Comparative data between PD and HD during pregnancy remain limited due to the rarity of cases and the predominance of retrospective reports. However, observational studies and case series suggest that intensive HD (≥ 36 h/week) is associated with higher birth weights and longer gestation [7]. PD, while less commonly used during pregnancy, offers benefits such as hemodynamic stability, continuous solute clearance, and preservation of residual renal function—factors that are independently associated with better fetal outcomes [6, 9]. A meta‐analysis by Li et al. (2023) [14] found no significant difference in live birth rates between PD and intensive HD, although PD was associated with fewer hospitalizations. Nonetheless, limitations in study size and heterogeneity preclude definitive conclusions, highlighting the need for more robust comparative data.

Pregnancy outcomes reported in patients on peritoneal dialysis versus hemodialysis are summarized in Table 1

**TABLE 1:** Pregnancy outcomes on peritoneal dialysis (PD) literature review.

Author (year)	PD status during pregnancy	Patient age	Dialysis management	Pregnancy outcome	Transporter type
Lim et al. [3]	Continued (CAPD)	42	CAPD; 4 exchanges/day; Kt/V 1.93–2.73; UF 500–1500 mL/day	C‐section at 36 weeks	Not reported
Chang et al. [12]	Continued (Tidal APD)	Not Reported	Tidal PD; intensified clearance	Successful delivery (gestation not reported)	Not reported
Tuncer [15]	Continued (CAPD)	25	CAPD; treated peritonitis; no modality change	Healthy term delivery	Not reported
Smith WT et al. [10]	Transitioned to HD	Not reported	HD 6×/week; PD catheter preserved	Term infant; resumed PD postpartum	Not reported
Nivelle et al. [5]	Transitioned to HD	Not reported	Switched to intensive HD in 2nd trimester	Successful delivery	Not reported
Alhwiesh et al. [9]	Continued (Tidal APD)	Various	8 patients; Tidal APD	All live births; no major complications	Not reported
Verrismo [16]	Continued (APD)	22	Switched to APD at 12 weeks	Healthy baby at 35 weeks	Not reported
Choi et al. [17]	Continued (PD)	Not reported	Maintained PD throughout pregnancy	Successful delivery	Not reported
Batarse et al. [18]	Continued (CAPD)	Not reported	CAPD; adjusted PD prescription	Near full‐term delivery	Not reported
Chou et al. [13]	Continued (CAPD)	Not reported	Managed hemoperitoneum conservatively	Healthy baby	Not reported

In summary, our case adds to the growing literature demonstrating that PD, when managed thoughtfully, can be safely resumed postpartum. Preservation of the PD catheter was a proactive decision based on shared decision‐making, anticipating the patient's desire to resume PD postpartum. This aligns with best practices in individualized dialysis planning during pregnancy. Key enablers include catheter preservation, favorable peritoneal kinetics, residual kidney function, and patient preference. These insights support a more individualized and flexible approach to dialysis management in pregnant ESRD patients—one that considers the potential for PD continuation or reinitiation when clinically appropriate.

This case illustrates the safety and practicality of resuming peritoneal dialysis postpartum in patients who temporarily switch to hemodialysis during pregnancy. Successful outcomes rely on patient‐centered care, preservation of the PD catheter, and coordinated multidisciplinary management. As more women of childbearing age require dialysis, adopting flexible, individualized approaches to dialysis modality before and after pregnancy becomes increasingly important. Key clinical considerations include maintaining catheter patency, confirming preserved peritoneal membrane function through PET, assessing residual urine output, and incorporating patient preferences. These factors highlight the importance of establishing protocols that actively evaluate the feasibility of reinitiating PD postpartum, rather than defaulting to permanent HD transition. Further prospective studies and clinical guideline development are needed to support evidence‐based decision making in this area.

## Author Contributions

**Ahmad Matarneh:** conceptualization, data curation, writing – original draft, writing – review and editing. **Sundus Sardar:** writing – original draft, writing – review and editing. **Omar Salameh:** writing – original draft, writing – review and editing. **Abdelrauof Akkari:** writing – original draft, writing – review and editing. **Umar Farooq:** writing – original draft, writing – review and editing. **Navin Verma:** writing – original draft, writing – review and editing. **Nasrollah Ghahramani:** supervision, writing – original draft, writing – review and editing. **Ronald Miller:** supervision, writing – original draft, writing – review and editing.

## Consent

Written informed consent was obtained from the patient to publish this report in accordance with the journal's patient consent policy.

## Conflicts of Interest

The authors declare no conflicts of interest.

## Data Availability

Data included in this manuscript will be available upon a reasonable request.
