# Correction to ‘Resumption of Peritoneal Dialysis Postpartum Following Pregnancy in an ESRD Patient: A Case Report and Literature Review’

**DOI:** 10.1002/ccr3.71935

**Published:** 2026-01-26

A. Matarneh, S. Sardar, O. Salameh, et al., “Resumption of Peritoneal Dialysis Postpartum Following Pregnancy in an ESRD Patient: A Case Report and Literature Review,” *Clinical Case Reports* 13, no. 10 (2025): e71266, 10.1002/ccr3.71266.

In the published version of this article, errors were identified in the reference list limited to bibliographic citation details, including incorrect or incomplete author names, journal titles, publication years, volume or issue numbers, and page ranges. During the correction process, the entire reference list was reviewed against the original source publications and corrected where necessary to ensure accuracy and consistency with the cited literature. No references were added or removed, and no changes were made to the in‐text citations, scientific content, data interpretation, or conclusions of the article. The authors apologize for these errors

Reference #2

In Published version:

G. B. Piccoli, G. Cabiddu, R. Attini, et al., “Pregnancy in CKD: Whom Should We Follow and Why?,” *Nephrology, Dialysis, Transplantation* 29, no. 6 (2014): 1134–1140, https://doi.org/10.1093/ndt/gfu022.

In new version:

Piccoli GB, Fassio F, Attini R, Parisi S, Biolcati M, Ferraresi M, Pagano A, Daidola G, Deagostini MC, Gaglioti P, Todros T. “Pregnancy in CKD: Whom Should We Follow and Why?” *Nephrology Dialysis Transplantation* 27, no. 3 (2012): iii111–iii118.

Reference #4

In Published version: DOI is wrong

I. Giatras, D. P. Levy, F. D. Malone, J. A. Carlson, P. Jungers, and M. D. Lindheimer, “Pregnancy During Dialysis: Case Report and Management Guidelines,” *American Journal of Kidney Diseases* 31, no. 5 (1998): 770–775, https://doi.org/10.1053/ajkd.1998.v31.pm9589436.

In new version:

Giatras I, Levy DP, Malone FD, Carlson JA, Jungers P. “Pregnancy During Dialysis: Case Report and Management Guidelines.” *Nephrology, Dialysis, Transplantation: Official Publication of the European Dialysis and Transplant Association‐European Renal Association*. 13, no. 12 (1998): 3266–3272.

Reference #5

In Published version: Listed authors wrong

L. Nivelle, T. Lobbedez, M. Rabilloud, and M. Buchler, “Changing Dialysis Modality During Pregnancy: A Case Report,” *Peritoneal Dialysis International* 38, no. 5 (2018): 377–379, https://doi.org/10.3747/pdi.2018.00054.

In new version:

Shaw J, Katopodis C, Hladunewich MA, Ryz K. “Changing Dialysis Modality During Pregnancy: A Case Report,” *Peritoneal Dialysis International* 38, no. 6 (2018): 456–458.

Reference #6

In Published version:

S. Hou, “Pregnancy in Dialysis Patients: Is the Evidence Strong Enough to Lead Us to Change Our Counseling Policy?,” *Nature Reviews. Nephrology* 6, no. 6 (2010): 333–339, https://doi.org/10.1038/nrneph.2010.56.

In new version:

Piccoli GB, Conijn A, Consiglio V, Vasario E, Attini R, Deagostini MC, Bontempo S, Todros T. “Pregnancy in Dialysis Patients: Is the Evidence Strong Enough to Lead Us to Change Our Counseling Policy?” *Clinical Journal of the American Society of Nephrology*. 5, no. 1 (2010): 62–71.

Reference #7

In Published version:

M. Hladunewich, D. Schatell, R. M. Lindsay, P. E. Pergola, and C. T. Chan, “Intensive Hemodialysis and Pregnancy Outcomes in North America,” *American Journal of Kidney Diseases* 63, no. 2 (2014): 329–335, https://doi.org/10.1053/j.ajkd.2013.08.006.

In new version:

Hladunewich MA, Hou S, Odutayo A, Cornelis T, Pierratos A, Goldstein M, Tennankore K, Keunen J, Hui D, Chan CT. “Intensive Hemodialysis Associates With Improved Pregnancy Outcomes: A Canadian and United States Cohort Comparison,” *Journal of the American Society of Nephrology*. 25, no. 5 (2014): 1103–1109.

Reference #9 linked google scholar wrong

In Published version:

A. Alhwiesh, M. Al Sulaiman, A. Alhassan, et al., “Pregnancy in Peritoneal Dialysis Using Tidal Automated PD: Largest Case Series,” *Archives of Nephrology and Urology* 7 (2024): 21–28.

In new version:

Link: https://www.fortunejournals.com/articles/pregnancy‐in‐peritoneal‐dialysis‐using‐tidal‐automated‐peritoneal‐dialysis‐largest‐case‐series‐single‐‐center‐experience.html

Reference #11 DOI is wrong

In Published version:

I. Okundaye, P. Abrinko, and S. Hou, “Registry of Pregnancy in Dialysis Patients,” *American Journal of Kidney Diseases* 31, no. 5 (1998): 766–773, https://doi.org/10.1053/ajkd.1998.v31.pm9589435.

In new version:

Okundaye I, Abrinko P, Hou S. “Registry of Pregnancy in Dialysis Patients,” *American journal of kidney diseases*. 31, no. 5 (1998): 766–773.

Reference #12

In Published version:

J. W. Chang, H. L. Tsai, W. J. Ko, C. H. Lee, C. W. Yang, and M. S. Wu, “Pregnancy in a CAPD Patient: Role of Tidal Peritoneal Dialysis,” *Peritoneal Dialysis International* 22, no. 4 (2002): 522–523.

In new version:

Hu X, Ren H. “Successful Pregnancy and Delivery Case in a Peritoneal Dialysis Patient: A Case Report and Review of Literature,” *Medicine*. 105, no. 2 (2026): e47103.

Reference #13

In Published version:

C. Y. Chou, Y. F. Lin, C. K. Yang, and H. C. Fang, “Hemoperitoneum complicating peritoneal dialysis in pregnancy,” *American Journal of Kidney Diseases* 47, no. 6 (2006): e89–e93, https://doi.org/10.1053/j.ajkd.2006.02.178.

In new version:

Chou CY, Ting IW, Hsieh FJ, Lee CN. “Haemoperitoneum in a Pregnant Woman With Peritoneal Dialysis,” *Nephrology Dialysis Transplantation*. 21, no. 5 (2006): 1454–1455.

Reference #14

In Published version:

Y. Li, Y. Zhang, J. Lin, et al., “Pregnancy Outcomes in Dialysis Patients: a Systematic Review and Meta‐Analysis,” *Med4* 2, no. 3 (2023): e56, https://doi.org/10.1002/med4.56.

In new version:

Fan L, Yang X, Shen H, Chen M, Zhang H, Ni Z, Lin H, Yang H, Chen Q, Chen H, Jiang G. “A Prospective, Randomized, Multicenter, Open‐Label Trial Comparing Survival in Subjects Receiving Peritoneal Dialysis or Conventional In‐Center Hemodialysis,” *Medicine Advances*. 2, no. 2 (2024): 112–122.

